# Combining mouse mammary gland gene expression and comparative mapping for the identification of candidate genes for QTL of milk production traits in cattle

**DOI:** 10.1186/1471-2164-8-183

**Published:** 2007-06-20

**Authors:** Micha Ron, Galit Israeli, Eyal Seroussi, Joel I Weller, Jeffrey P Gregg, Moshe Shani, Juan F Medrano

**Affiliations:** 1 Department of Quantitative and Molecular Genetics, Agricultural Research Organization, The Volcani Center, Bet Dagan, 50-250, Israel; 2 Department of Pathology and M.I.N.D Institute, University of California, Davis, 2825 50th Street, Sacramento, CA 95817, USA; 3 Department of Animal Science, University of California, Davis, One Shields Ave. Davis, CA 95616, USA

## Abstract

**Background:**

Many studies have found segregating quantitative trait loci (QTL) for milk production traits in different dairy cattle populations. However, even for relatively large effects with a saturated marker map the confidence interval for QTL location by linkage analysis spans tens of map units, or hundreds of genes. Combining mapping and arraying has been suggested as an approach to identify candidate genes. Thus, gene expression analysis in the mammary gland of genes positioned in the confidence interval of the QTL can bridge the gap between fine mapping and quantitative trait nucleotide (QTN) determination.

**Results:**

We hybridized Affymetrix microarray (MG-U74v2), containing 12,488 murine probes, with RNA derived from mammary gland of virgin, pregnant, lactating and involuting C57BL/6J mice in a total of nine biological replicates. We combined microarray data from two additional studies that used the same design in mice with a total of 75 biological replicates. The same filtering and normalization was applied to each microarray data using GeneSpring software. Analysis of variance identified 249 differentially expressed probe sets common to the three experiments along the four developmental stages of puberty, pregnancy, lactation and involution. 212 genes were assigned to their bovine map positions through comparative mapping, and thus form a list of candidate genes for previously identified QTLs for milk production traits. A total of 82 of the genes showed mammary gland-specific expression with at least 3-fold expression over the median representing all tissues tested in GeneAtlas.

**Conclusion:**

This work presents a web tool for candidate genes for QTL (cgQTL) that allows navigation between the map of bovine milk production QTL, potential candidate genes and their level of expression in mammary gland arrays and in GeneAtlas. Three out of four confirmed genes that affect QTL in livestock (*ABCG2*, *DGAT1*, *GDF8, IGF2*) were over expressed in the target organ. Thus, cgQTL can be used to determine priority of candidate genes for QTN analysis based on differential expression in the target organ.

## Background

Many studies have found segregating quantitative trait loci (QTL) for milk production traits in different dairy cattle populations [reviewed by Khatkar et al., [1] and Polineni et al., [2]; [3]]. However, even for relatively large effects with a saturated marker map, the confidence interval (CI) for QTL location by linkage analysis spans tens of map units, or hundreds of genes. Many studies have shown that CI for QTL can be further reduced by application of linkage disequilibrium (LD) mapping [e.g. [4]]. This requires genotyping additional polymorphisms within the CI, generally single nucleotide polymorphisms (SNP). Although any marker within the CI can be used for LD mapping, it is reasonable to start with polymorphisms embedded within genes that are likely candidates for the QTL [5].

Genes within the confidence interval that have some physiological relevance to the trait will be considered primary candidates for the QTL. For example, Grisart et al. [6] concluded that the gene *DGAT1*, which is involved in triglyceride synthesis, is the causative gene for the QTL affecting milk fat on BTA14. Wayne and McIntyre [7] have suggested combining mapping and arraying as an approach to identify candidate genes. Thus, gene expression analysis can bridge the gap between fine mapping and quantitative trait nucleotide (QTN) determination by revealing regulatory variation in genes for complex traits [8-10]. Specific tissue of origin of expressed sequence tags (EST), tissue-specific, and tissue-selective gene expression may also indicate a potential role of genes in regulation of QTL [9,11]. Microarrays have been used to measure relative expression, and thus identify candidate genes responsible for QTL [12,13]. Additional criteria may be proposed to select QTL candidate genes as follows:

1. Genes are preferentially expressed in organs related to the quantitative trait, i.e. the mammary gland for milk production traits.

2. Genes are preferentially expressed in developmental stages related to the phenotype, i.e. at the onset of lactation for milk production traits.

Su et al. [11] measured the mouse and human protein-encoding transcriptomes, and used them to profile a panel of human and mouse tissues in gene atlas, therby providing a resource to address tissue-specific expression. The USDA has announced a project to construct a bovine gene atlas using gene expression analysis data derived from 100 tissues of the cow genome.

Although a cDNA microarray resource enhanced for bovine mammary gland has been developed [14], and a bovine oligonucleotide DNA microarray was used to identify estrogen-responsive genes in the bovine mammary gland [15], there is no information available on bovine mammary gland gene expression at different stages of development. However, detailed studies examining gene expression in the mammary gland during, puberty, pregnancy, lactation, and involution have been carried out in the mouse [16-18]. In these studies the same microarray platform (Affymetrix MG-U74Av2) was used in similar mouse inbred lines resulting in a high level of replication, and consequently in one of the best data resources on mammary tissues gene expression relevant to milk production traits. In addition, the high conservation of gene order in mammals enables comparative mapping to be a useful approach linking clusters of genes with similar function between mouse and cattle [19]. We have utilized murine mammary gland gene expression in combination with bovine QTL mapping data to create a web tool (cgQTL) that compiles all the available information to aid in the identification of candidate genes for QTL of milk production traits in dairy cattle.

## Results

### Analysis of gene expression from three experiments

**Figure 1:**
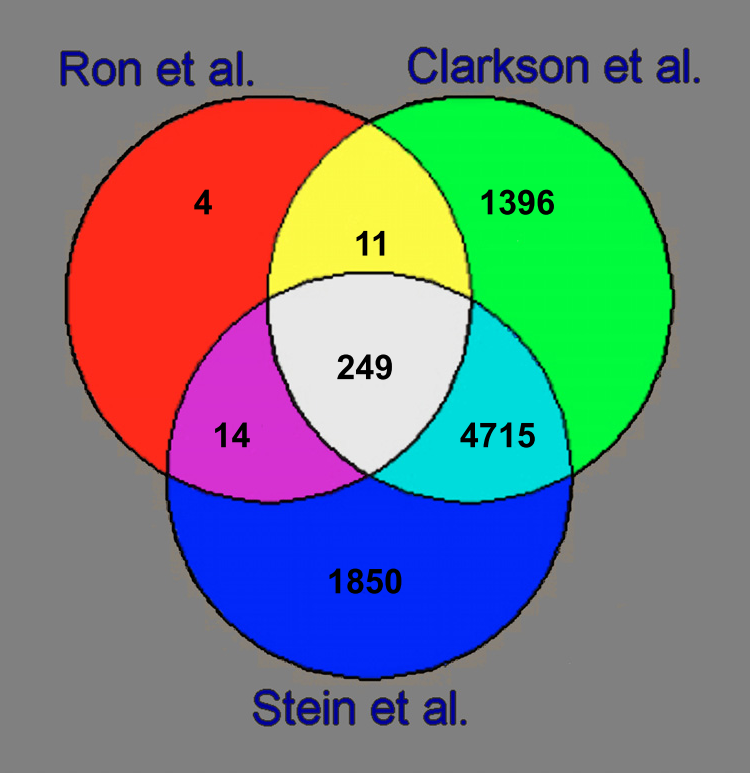
Venn diagram of the three microarray experiments.

A total of 278 probe sets with significant differential expression across the four stages in the current study were obtained using ANOVA. The Venn diagram in figure 1 shows that two other microarray experiments [16,18] using the same statistical analysis found thousands of significant probe sets (Table 1). Observed and expected frequencies for probe sets discovery in the three experiments alone and in the two experiments of Clarkson and Stein are given in Table 2. The Chi-squared value for joint discovery of probe sets in Clarkson and Stein experiments was 2834. Of the 278 significant probe sets in the current study, 249 (90%) were common to all three experiments. The expected value was only 77 and the Chi-squared value was 3240.

**Table 1:** Experimental design of the three microarray experiments

Experiment^1^	Mouse line	Stage	N. of time points within a stage	N. of arrays
C	C57/Bl	Puberty	1	2
		Pregnancy	3	6
		Lactation	3	6
		Involution	5	10
		Total	12	24
R	C57BL/6J	Puberty	1	3
		Pregnancy	1	2
		Lactation	1	2
		Involution	1	2
		Total	4	9
S	Balb/C	Puberty	2	6
		Pregnancy	7	4
		Lactation	3	9
		Involution	5	15
		Total	17	34

^1^C = Clarkson et al. (16); R = Ron et al. (17); S = Stein et al. (18)

**Table 2:** Observed and expected frequencies of significant genes for two and three microarray experiments.

Experiments compared^1^	Significance^1^	Observed	Expected	Chi-squared^2^
C & S	C & S	4964	3483	2834
	C only	1407	2888	
	S only	1864	3345	
	Neither	4253	2772	
C & S & R	C & S & R	249	77	3240
	C & S	4964	3483	
	C & R	11	64	
	S & R	14	74	
	C only	1407	2888	
	S only	1864	3345	
	R only	4	62	
	None	4249	2710	

^1^C = Clarkson et al. (16); R = Ron et al. (17); S = Stein et al. (18)

^2^Highly significant by any criterion

**Figure 2:**
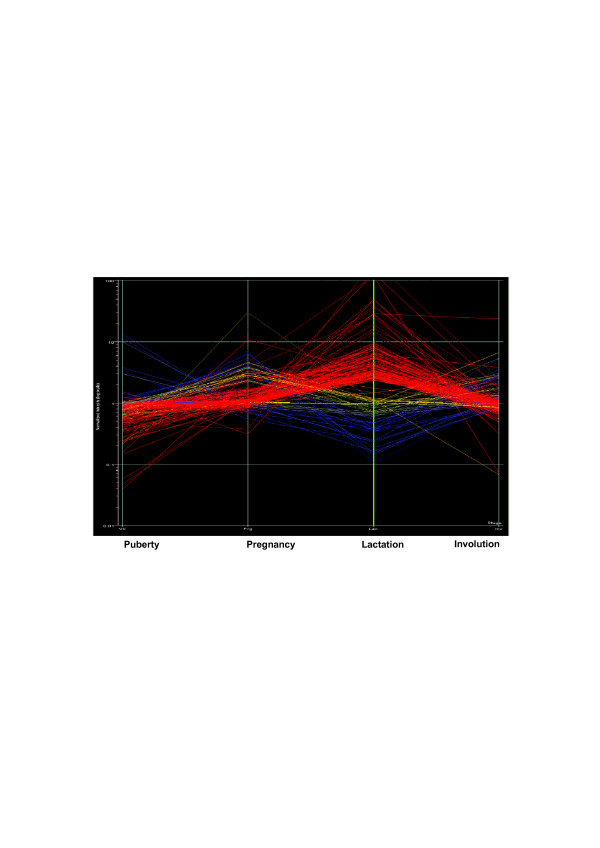
212 significant differentially expressed genes.

**Figure 3:**
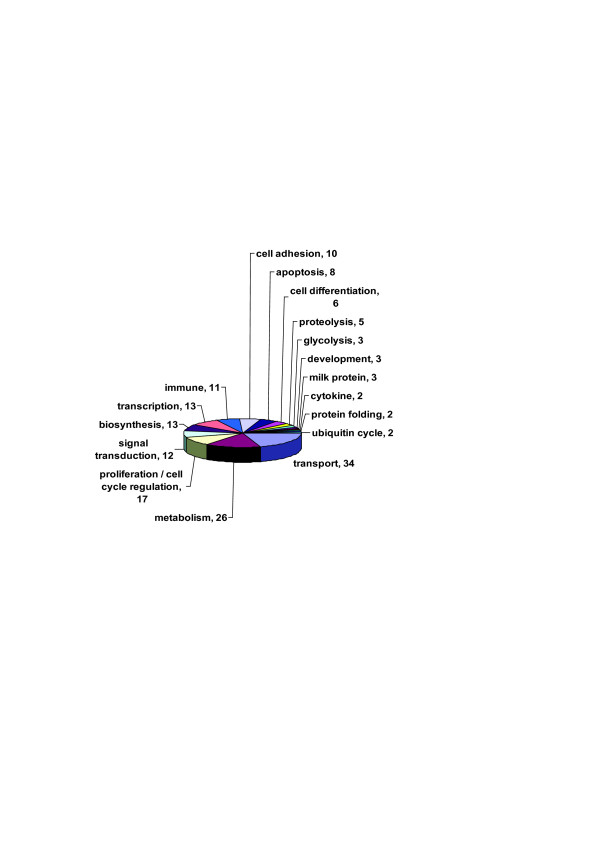
Distribution of significant genes by functional categories.

The 249 probe sets were assigned to 212 bovine genes. Three clones containing repeats, 21 unknown probe sets and 13 redundant probes were excluded. The expression profile of the 212 genes is presented in Figure 2. The distribution of genes by functional categories is presented in Figure 3. The majority of genes were assigned to metabolism, immune cascade, transport, regulation of transcription and signal transduction functional categories.

**Figure 4:**
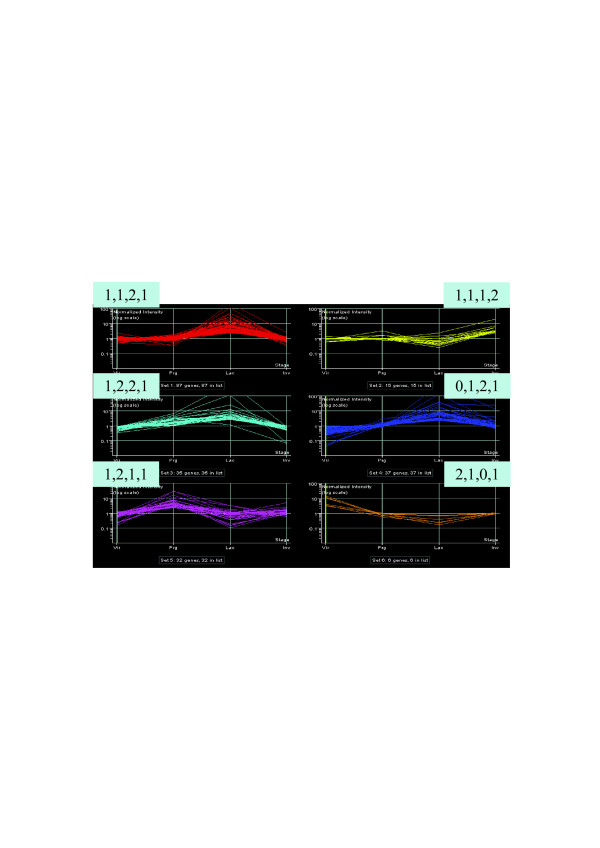
K-means clustering with expression profile signatures.

**Figure 5:**
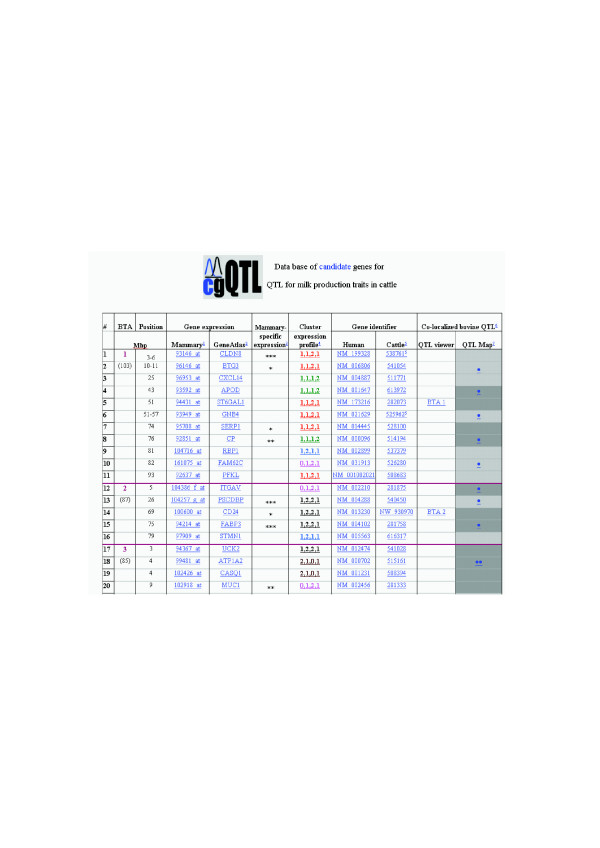
cgQTL data base.

The explained variance of expression of the 212 genes varied from 51 to 62 % using 5 to 15 K means clustering. Six K-means classifications were determined as the optimal number of clusters based on highest explained variance and minimum redundancy between similar clusters. The clustering explained 53% of the variance of expression. Each one of the six clusters was designated with its unique expression profile signature across stages (puberty, pregnancy, lactation, involution) and is presented in Figure 4. There were 87 over expressed genes at lactation only (1,1,2,1), and 72 additional over expressed genes at lactation with differential expression in other stages (1,2,2,1 and 0,1,2,1). Among the typical genes of lactation stage are the milk protein and biogenesis genes (Table 3). Fifteen genes were upregulated at involution only (1,1,1,2) such as *CXCL14 *and *SLPI*, involved in chemotaxsis and immune response processes (20, 21). Thirty-two genes were upregulated at pregnancy only (1,2,1,1). For example, *CRABP2 *and *CSRP1*, which are involved in development and cellular differentiation [22,23] are included in this group. The smallest group of six genes including myosin (*MYL1*) were upregulated at puberty, and downregulated at lactation (2,1,0,1). Of the 212 significant genes, 82 (39%) showed mammary gland-specific expression (Figure 5). Of these, 73 genes were upregulated at the onset of lactation, and the remaining genes were up regulated at pregnancy (7 genes) and at involution (2 genes).

**Table 3:** K-means clustering of gene expression profiles along four developmental stages (Puberty, Pregnancy, Lactation, Involution)

Set	N. of genes	Expression profile	Expression pattern^1^	Representative genes
1	87	1,1,2,1		*CSNd*, *PTHLH*, *B4GALT1*
2	15	1,1,1,2		*CTSS*, *LEP*, *MGP*, *HPDG*, *SLP1*
3	35	1,2,2,1		*CD24, FABP3, UCK2, CD320*
4	37	0,1,2,1		*CSNk*, CSNd, *LTF, LALBA*, *BTN1A1*, *XDH*, *MFGE8*
5	32	1,2,1,1		*STMN1*, *CRABP2*, *RELN*, *PHLDA1*, *CSRP1*
6	6	2,1,0,1		*INF4*, *Cox8b*, *ENO3*, *MYL1*, *ATP1A2*,*CASQ1*

^1^Mean expression pattern of a cluster of genes

### Comparative mapping

The ongoing effort for annotation of the bovine genes on the draft sequence allows identification of many bovine orthologs of the murine genes represented by the probe sets of MG-U74Av2. However, for many genes the chromosomal location is still unknown. Larkin et al. [24] reported about 90% accuracy in prediction of cattle chromosome locations based on the human genome. Thus we used the human sequence of orthologous genes to predict the location of bovine genes with unknown chromosomal location.

### cgQTL data base

**Figure 6:**
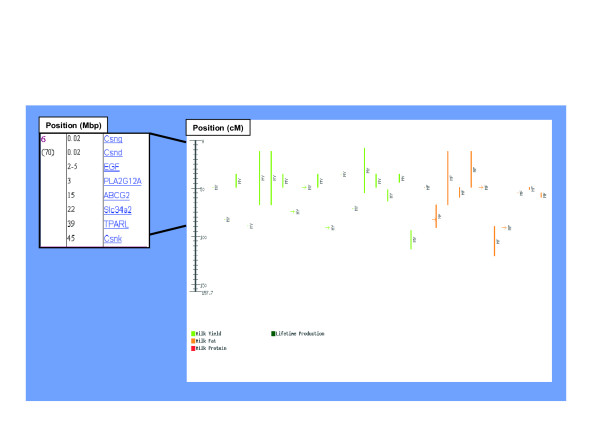
Co-localization of QTL map of BTA6 and candidate genes.

The structure of the cgQTL navigator is presented in Figure 5. The navigator presents an HTML list of candidate genes by bovine autosomes for QTL for milk production traits. Genes with mammary gland-specific expression are denoted with one to three stars indicating their level of specificity. QTL for milk production traits were found in all bovine autosomes and are referred through two dedicated data bases, QTL viewer and QTL map. In the latter, QTL are denoted with one to three stars in relation to the number of literature references where the QTL was reported. Candidate genes for QTL were found in all but two autosomes. Number of candidate genes for QTL per chromosome ranged from 1 to 17. As a demonstration, a co-localization of eight candidate genes with the QTL region for milk production traits on BAT6 is presented in Figure 6. The gene *ABCG2 *which was determined as the causative gene for this QTL appears on the list. The expression profile of this gene at pregnancy and lactation was in accordance to that in cattle [8].

## Discussion

Three data sets with Affymetrix MG-U74Av2 mammary gland arrays were compared along four developmental stages at puberty, pregnancy, lactation and involution. The additional data set of Rudolph et al. [25] consisted of the same developmental stages excluding puberty, and thus was not analyzed in this study. The mammary gland at the four selected developmental stages consists of different cell populations and processes, and therefore, most of the genes will show differential expression if sufficient number of replicates are included in the analysis. Two microarray experiments [16,18] used 75 animal replicates, while the current study [17] used nine animal replicates. The same filtering, normalization, and analysis of variance was applied on data of the three experiments. The first two studies found thousands of significant genes, as compared to hundreds in the current study. This is apparently due to the major difference in the number of replicates. Among the mammary gland-specific probe sets only four have unknown function (100949_at, 160549_at, 103343_at, 93479_at). Analysis of these genes may reveal their important role in the mammary gland.

Based on comparative mapping nearly all of the differentially expressed genes were mapped to their bovine chromosomal positions. The rate of chromosomal breakage during mammalian evolution is doubled in the rodent lineage compared to the cattle lineage [26]. Therefore we based the prediction of cattle chromosome locations on the human genome and the detailed cattle-human comparative maps [27]. The genes were encoded for expression (0, 1, 2) in each of the four developmental stages, and were clustered to six expression profiles, out of the 81 possible profile combinations (3^4 ^= 81). These clusters correspond to genes showing upregulation uniquely at pregnancy or lactation or involution, and differential expression at combinations of stages involving puberty, pregnancy, and lactation. Rudolph et al. (25) presented a thorough study of expression profiling of secretory activation of 1358 genes using 72 clusters, and Clarkson et al. [16] analyzed the expression profiling of 6796 genes using 35 clusters.

Our cgQTL web tool [28] allows for navigation between the map of bovine milk production QTL, the overlaid candidate genes and the visual presentation of their expression in the mammary gland array and in GeneAtlas. Genes that are mammary gland-specific are indicated. To truly infer that a differentially expressed gene, which is located in a QTL, is a candidate gene for a trait being studied, information on the cis- or trans-regulation of the gene needs to be presented [29]. However, such data is not available for *Bos taurus*. Therefore, our cgQTL tool was developed to provide the best current compilation of information to identify candidate genes for milk production in cattle, and will continue improving as we add more sources of information.

Figure 6 shows the co-localization of candidate genes with QTL on BTA6 affecting milk production traits. *ABCG2 *is listed as one of eight candidate genes. Of the two identified genes that have been proven to affect milk production traits in dairy cattle, *ABCG2 *and *DGAT1 *on BTA 6 and 14, respectively [6,8], only the former gene was upregulated in the mammary gland at the onset of lactation. Both genes that have been proven to affect muscularity in sheep and pigs, myostatin (*GDF8*) and *IGF2*, showed differential expression in muscle [30,31]. Thus, three out of four confirmed genes that affect QTL in livestock were over expressed in the target organ. Therefore, cgQTL can be used to determine priority of candidate genes for QTN analysis based on differential expression in the target organ. Nevertheless, the MG-U74Av2 array has a limited set of probes representing less than one-half of the mouse genome. Thus, future studies with arrays covering the entire repertoire of mouse genes will be needed to update the current web tool [32].

The argument could be made against the feasibility of using mouse data to make inferences to bovine mammary gland gene expression. However, our objective in this work was to create a tool (cgQTL) that utilizes the best available sources of information, such as mouse gene expression data during mammary development and bovine QTL mapping data to develop hypotheses on potential candidate genes underlying QTL. This is a comparative approach to integrate information in the difficult task of identifying candidate genes. We envision that in the future the mouse gene expression data used in cgQTL will be complemented with bovine gene expression data from experiments using cDNA microarrays [14], bovine oligonucleotide arrays (24,000 probes; [33]) and with the Affymetrix GeneChip^® ^arrays (23,000 transcripts). cgQTL navigator will also be linked to the bovine GeneAtlas which will be developed using gene expression analysis derived from 100 bovine tissues. Likewise, cgQTL may be expanded for QTL of reproduction [34,35]. Various resources for the study of ovarian transcriptome may be integrated into cgQTL to predict candidate genes for QTL related to reproductive traits [12,36-39].

## Conclusion

Here we present a web tool for candidate genes for QTL (cgQTL) that allows navigation between the map of bovine milk production QTL, the overlaid candidate genes and the visual presentation of their expression in the mammary gland array and in GeneAtlas. Three out of four confirmed genes that affect QTL in livestock were over expressed in the target organ. Thus, cgQTL can be used to determine priority of candidate genes for QTN analysis based on differential expression in the target organ.

## Methods

### Mouse mammary tissue

Mammary gland no. 4 (inguinal) fat pads were harvested from three C57 female mice at puberty (6 wks), two females at pregnancy (14 d), two females at lactation (10 d) and two females at postlactational involution (4 d). The tissue was placed in RNAlater (Ambion Inc.) and kept at -80°C. RNA was isolated using Trizol (GibcoBRL). Conception date was designated as the day a vaginal plug was observed. Pregnancy at 14 d was confirmed by assessment of developmental stage of embryos at autopsy [40]. The characteristic feature of 14 d embryos is that the individual fingers are separated in the forefoot plate, but not in the hindfoot plate.

### Experimental design and microarray

The design of three microarray experiments with number of animal replicates for each developmental stage is presented in Table 1. Fragmented cRNA was prepared (15 Mg) and hybridized overnight to murine MG-U74Av2 (12,488 probes) Affymetrix GeneChip arrays according to the manufacturer's protocols (Affymetrix, Santa Clara, CA). Arrays were processed at the University of California Davis School of Medicine Microarray Core Facility. The complete dataset is publicly available [41].

### Filtering and normalization

The data was incorporated into GeneSpring 6.2 (Silicon Genetics, CA). Transcripts were removed if mean signal intensities were not above 20 signal units in at least one out of the four stages. Per chip and per gene normalizations have been applied following Affymetrix' guidelines.

### Public mouse mammary gland arrays

Microarray experiments with mouse mammary gland and the same Affymetrix Genechip array MG-U74Av2 are available at public data bases [9,36]. RNA was extracted from mammary gland of 51 C57 black and 24 Balb/C females, respectively. The tissues were from mammary gland at four developmental stages as define above (Table 1). Samples from early, mid and late stage were combined. Filtering and normalization using GeneSpring software were applied as above.

### Analysis of variance

Analysis of variance was applied to each of the three experiments, separately, using the parametric test with all available error estimates in GeneSpring, and FDR of 0.05 [42].

### Venn diagram

The Venn diagram in GeneSpring was applied to the three lists of significant genes resulting from ANOVA for the different experiments.

### Clustering and coding of expression profiles

The expression data from the current study for 212 significant genes were subjected to a range of 5 to 15 K-means classifications using Pearson correlations [43]. The optimal number of clusters was determined empirically based on highest explained variance and minimum redundancy between similar clusters. We adopted the procedure of Rudolph et al. [25] to code the mean expression of a cluster at each stage as flat, decrease, and increase and converted it to numerical representation as follows: "1" indicates no change between 0.5 and 2.0 fold change, and "0" and "2" indicate fold change ≤ 0.5 and ≥ 2 for down and up regulation, respectively. Thus a unique array of four digits represents the expression profile of each cluster along the developmental stages of puberty, pregnancy, lactation and involution.

### Mammary gland-specific expression

Mammary gland-specific expression of a gene was denoted by a single star in the navigator if expression was between 3 to 10 fold from median representing all tissues tested in GeneAtlas [44]. Two stars for the range of 10 to 30 fold, and three stars for > 30 fold expression.

### Functional category analysis

Genes were assigned to 17 different categories according to Gene Ontology Consortium (GO) [45]. In the case of insufficient GO information, the conserved domain of the protein, GO information on orthologous genes and data from published literature were used to classify the gene into a functional category.

### Comparative mapping

To associate the probe sets (targets) of MG-U74Av2 with genes, sequences of the mouse targets were masked for repetitive elements [46] and *Blast *searched against mouse Reference Sequences (RefSeqs). The target was considered unknown if the *Blast *search resulted in no significant similarity. To identify bovine genes orthologous to these targets and to predict their map locations in *Bos taurus*, the mouse RefSeqs were *Blast *searched against human genome build (nr database) and the associated human RefSeqs were used to *Blast *search the cow genome. When such search indicated a bovine gene with unknown mapping data, the human genes adjacent to the gene in the query, were similarly searched in the cow genome in order to infer the bovine location based on synteny. In the body of text, data base and figures, gene/transcripts are named according to the Mouse Genome Database [47].

### Position of genes on genetic and physical maps

To relatively locate genes to the critical interval of the QTL for milk production traits, the alignment of the physical and the genetic maps was used [48].

### Archived data

The complete dataset, including the 9 raw data .cel files fulfilling MIAME criteria are publicly available [41].

### Statistical analysis

Expected frequencies for eight and four combinations of significance under the assumption of random assortment of probe sets among the three and two experiments, respectively were computed based on the total number of 12,488 transcripts, and the number of transcripts with statistical significance in each of the three and two experiments. Significance of the deviation from random assortment was tested by Chi-squared. The chi-squared values were computed for the results of each experiment separately. In this case the chi-squared test has only a single degree of freedom.

### cgQTL data base

We present a web tool for candidate genes for QTL, cgQTL [28] that allows navigation between the map of bovine milk production QTL, the overlaid candidate genes common to the three experiments, and the visual presentation of their expression in the mammary gland array and in GeneAtlas. The data base allows searches for specific genes. For each gene links are available for expression in the mammary gland array along four stages, expression in a variety of tissues of GeneAtlas, mammary gland-specific expression, expression profile signature, NCBI identifiers for human and cow, QTL viewer and QTL map. The expression profile signature is indicated for all genes classified to the same cluster.

## Authors' contributions

MR performed the microarray experiment, supervised the design of cgQTL and drafted the manuscript. GI analyzed the microarray data and built the data base under the bioinformatics guidelines of ES. JIW performed the statistical analysis and helped draft the manuscript. JPG did the hybridization of microarrays. MS supervised the design of the data base. JFM supervised the design of the microarray experiment and helped draft the manuscript. All authors read and approved the final manuscript.
